# Fish Oil Enriched in EPA, but Not in DHA, Reverses the Metabolic Syndrome and Adipocyte Dysfunction Induced by a High-Fat Diet

**DOI:** 10.3390/nu13030754

**Published:** 2021-02-26

**Authors:** Roberta Dourado Cavalcante da Cunha de Sá, Jussara de Jesus Simão, Viviane Simões da Silva, Talita Mendes de Farias, Maysa Mariana Cruz, Vitor Jacó Antraco, Lucia Armelin-Correa, Maria Isabel Alonso-Vale

**Affiliations:** Department of Biological Sciences, Institute of Environmental Sciences, Chemical and Pharmaceutical, Universidade Federal de São Paulo—UNIFESP, Diadema 09913030, Brazil; rdccunha@gmail.com (R.D.C.d.C.d.S.); jus_simao@hotmail.com (J.d.J.S.); ss.vivi95@gmail.com (V.S.d.S.); talitamfarias@hotmail.com (T.M.d.F.); maysamariana@gmail.com (M.M.C.); vitor_antraco@hotmail.com (V.J.A.); lucia.correa@unifesp.br (L.A.-C.)

**Keywords:** obesity, omega-3 fatty acids, insulin resistance, eicosapentaenoic acid, docosahexaenoic acid

## Abstract

This study aimed to investigate the effects of two commercially available fish oils (FOs) containing different proportions of two omega-3 fatty acids (FA), eicosapentaenoic acid (EPA) and docosahexaenoic acid (DHA), on the metabolic and endocrine dysfunctions of white adipose tissue resulting from obesity. Male C57BL/6J mice, 8 weeks old, received a control or high-fat diet (CO and HF groups, with 9% and 59% energy from fat, respectively) for 8 weeks. The next 8 weeks, the HF group was subdivided into HF, HF+FO/E (HF+5:1 EPA:DHA), and HF+FO/D (HF+5:1 DHA:EPA). Supplementation was performed by gavage, three times a week. All groups that received the HF diet had lower food and caloric intake, but a higher fat intake, body weight (BW) gain, glucose intolerance, and a significant increase in inguinal (ING), retroperitoneal (RP), and epididymal (EPI) adipose tissues when compared to the CO group. Additionally, HF and HF+FO/D groups showed insulin resistance, adipocyte hypertrophy, increased lipolysis and secretion of TNF-α, resistin and IL-10 adipokines by ING and RP adipocytes, and adiponectin only by the HF+FO/D group in ING adipocytes. All of these effects were completely reversed in the HF+FO/E group, which also showed partial reversion in BW gain and glucose intolerance. Both the HF+FO/E and HF+FO/D groups showed a reduction in ING and RP adipose depots when compared to the HF group, but only HF+FO/E in the EPI depot. HF+FO/E, but not HF+FO/D, was able to prevent the changes triggered by obesity in TNF-α, Il-10, and resistin secretion in ING and RP depots. These results strongly suggest that different EPA:DHA ratios have different impacts on the adipose tissue metabolism, FO being rich in EPA, but not in DHA, and effective in reversing the changes induced by obesity.

## 1. Introduction

Obesity is triggered by the interaction of several components, such as genetic, metabolic, behavioral, and environmental factors. The rapid increase in the prevalence of excess weight suggests that the behavioral and environmental features are the main responsible for this disease [1,2]. The abnormal or excessive accumulation of fat results from the imbalance between consumption and expenditure of calories, impairing health. Such imbalance is characterized by a greater intake of foods that have a high content of fat, cholesterol, sugar, and salt, as well as an increase in the consumption of processed foods and a significant reduction in physical activity [3,4]. Obesity gradually promotes a chronic low-grade inflammatory state and adipocytes begin to secrete pro-inflammatory cytokines, such as TNF-α, increasing macrophages infiltration in the adipose tissue.

It is well known that the conversion of arachidonic acid (ARA 20:4) into eicosanoids is an important inflammatory pathway. Several studies in humans have shown that omega-3 eicosapentaenoic acid (EPA 20:5) and docosahexaenoic acid (DHA 22:6) diet supplementation can alter the concentration of ARA, EPA, and DHA in blood cells. A supplementation with approximately 1:3 EPA:DHA ratio for 8 weeks not only reduces ARA but also increases EPA and DHA neutrophil content [5]. Subjects treated for 12 weeks with an approximately 4:1 EPA:DHA ratio presented a significant decrease in ARA:EPA ratio in plasma and mononuclear cells phospholipids [6]. Ingestion of a 1:1 EPA:DHA for 12 weeks led not only to an increase in EPA and relative DHA abundance in the plasma phospholipids, but also in the subcutaneous adipose tissue [7,8]. In mice, FO supplementation led to increased EPA+DHA in the gastrocnemius muscle [9]. It was hypothesized that EPA and DHA may compete to incorporate into the cell’s membrane phospholipids, leading to changes in membrane biophysical structure and the synthesis of downstream metabolites [10]. The different role of EPA and DHA in the resolution of an inflammatory process has also been investigated, as these FAs are precursors for different resolvins, biosynthesized lipid mediators, which act primarily in the immune system cells [11]. Besides, it has been demonstrated that EPA and DHA can activate GPR120, a G-protein coupled receptor, with different intensities of DHA, leading to a stronger response and mediating anti-inflammatory signaling, which reduces TNF-α pathway activation in macrophages [12]. In agreement with these findings, the visceral adipose tissue of mice fed with a high-fat diet and supplemented with EPA presented alterations in miRNAs expression and downregulation of several pathways involved in the inflammatory processes [13]. Besides all these sites of action, both EPA and DHA bind to PPARs and activate them in cell-based assays [14,15], leading to important alterations in gene expression in the adipose tissue.

Not surprising, dietary supplementation with FO, which has a high concentration of long-chain omega-3 polyunsaturated FA (omega-3 FA), including EPA and DHA, has been showing beneficial effects not only on the reduction of body mass gain triggered by a high-fat (HF) diet, but also on obesity and associated metabolic disorders, such as diabetes, on cardiovascular, inflammatory and autoimmune diseases [9,16,17,18,19,20]. These beneficial effects are attributed to the high concentration of omega-3 FA, including EPA and DHA [11,21,22,23].

However, the several studies on omega-3 FA differ in terms of model, time, and especially in the type and proportion of omega-3 FA. Our group has already shown the beneficial effects of FO rich in omega-3 FA, in the 5:1 EPA:DHA ratio, in the prevention and treatment of the metabolic syndrome and adipose cell disorders of diet-induced obese mice [16,17]. Taking into account that there are currently different proportions of FA in fish oils available and being commercialized, in the present work we aim to compare the effects on metabolism and cytokines of visceral and subcutaneous adipose tissue in mice fed with an HF diet and treated three times a week with two different ratios of EPA:DHA, namely: 5:1 EPA:DHA (27 mg EPA and 5 mg DHA), and 5:1 DHA:EPA (5 mg EPA and 25 mg DHA). For this purpose, we used two different FOs produced and commercialized by the same company (Naturalis). We hypothesize that these different ratios of EPA:DHA have different impacts on metabolic parameters altered by an HF diet-induced obesity. It is important to emphasize that both FOs used in this study are easily accessible to the population, who receive so much contradictory information about the benefits of omega-3 FA.

## 2. Materials and Methods

### 2.1. Animals, Diet, and Fish Oil Supplementation

**Figure 1:**
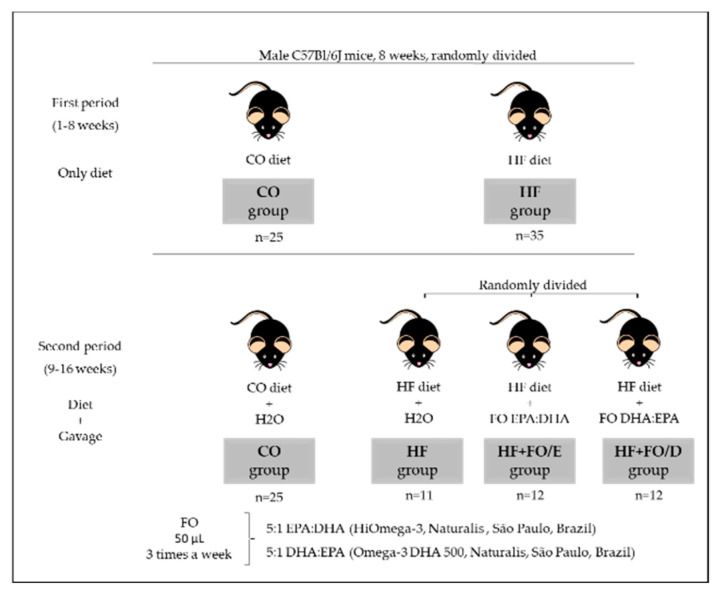
Experimental procedure. In the first period, the animals were randomly divided between CO or HF groups, which would receive the respective diets (CO: control diet and HF: high fat diet). In the second period, the CO group began to receive water by gavage, and the HF group was randomly divided into 3 groups: HF, which received water, HF+FO/E, which received FO (fish oil) containing a high concentration of EPA (eicosapentaenoic acid), and HF+FO/D receiving FO containing a high concentration of DHA. The gavage was performed 3 times a week, in a volume of 50 μL.

Male C57Bl/6J mice of 8 weeks from the Center for the Development of Experimental Models (CEDEME) of the Federal University of São Paulo were used. All procedures performed with animals were approved by the Research Ethics Committee of the Federal University of São Paulo, through the Ethics Committee on the Use of Animals (CEUA 7912040515). The diets were prepared in the laboratory, based on the AIN-93 (American Institute of Nutrition, 1993) [24]. After 1 week of adaptation the protocol was started, and lasted for 16 weeks. In the first 8 weeks (first period), the animals were randomly divided and received the diets, resulting in 2 groups: control (CO, 9% fat, 76% carbohydrates, and 15% proteins) and high fat (HF, 26% carbohydrates, 59% fat, and 15% proteins). In the following 8 weeks (second period), the HF group was randomly divided, and received (or not) FO (HiOmega-3, Naturalis, São Paulo, Brazil), resulting in 4 groups: CO, HF, HF+FO/E (HF diet supplemented with FO containing high concentration of EPA (550 mg/g) compared to DHA (EPA:DHA 5:1 ratio)) and HF+FO/D (HF diet supplemented with FO containing high concentration of DHA (500 mg/g) compared to EPA (DHA:EPA 5:1 ratio)). FO supplementation was performed by gavage, in a volume of 50 μL, 3 times a week, on Mondays, Wednesdays and Fridays, in the morning, between 9 and 10 am. The amount and composition of fat (indicated by the company) present in the two FOs used is showed in Table 1. The groups CO and HF received water in the same volume (Figure 1). The diet and FO dosage was chosen based on previous studies from our group [16,17,25,26].

### 2.2. Experimental Procedure

Once a week, the body weight and food intake were measured. After 16 weeks of the experimental protocol, 6 h fasted mice were anesthetized with isoflurane and killed by cervical dislocation. This procedure took place in the morning, between 9 and 10 a.m. Subcutaneous (inguinal—ING) and visceral (retroperitoneal—RP and epididymal—EPI) adipose fat depots, and liver were harvested and weighed.

### 2.3. Glucose and Insulin Tolerance Test

In the last week of the treatment protocol, the insulin tolerance test (ITT) and glucose tolerance test (GTT) were performed. Mice were fasted for 6 h and injected intraperitoneally with glucose (2 g/kg BW) to GTT, or insulin (0.75 UI/kg BW; Humulin R; Lilly, Indianapolis, IN, USA) to ITT. Tail-vein blood glucose levels were determined before and the 15, 30, 45, 60, and 90 min after glucose injection, and before and 3, 6, 9, 12, and 15 min after insulin injection using an OneTouch^®^ glucometer (Johnson and Johnson, Milpitas, CA, USA). Glucose concentration vs. time was plotted and the area under the curve was calculated for each animal, and glucose concentration vs. time was plotted and the glucose level lowering rate was calculated. The serum glucose disappearance constant (kITT) was estimated during the insulin tolerance test and calculated by the slope of the logarithm regression line of the glycemia against time during the first 3–15 min when the plasma glucose concentration falls linearly.

### 2.4. Blood Measurements

Triglycerides and total cholesterol were determined in the serum through commercial kits by colorimetric assays (Labtest Diagnostics, Minas Gerais, Brazil).

### 2.5. Adipocyte Isolation

Adipocyte isolation was performed as previously described [27] with slight modifications [17]. The adipocytes from the ING and RP adipose tissues were isolated by the technique of digestion of tissue by collagenase, described by Rodbell (1964), with some modifications to adapt the method to our laboratory conditions. In summary, the fats were incubated in 4 mL of digestive buffer (DMEM, HEPES 25 mM, 4% BSA, collagenase type II (Sigma Chemical, St. Louis, MO, USA) 1.25 mg/mL, pH 7.45), for about 60 min, at 37 °C, in a water bath, with orbital agitation (150 rpm). Then, the samples were filtered through a plastic sieve with a fine mesh (which retains tissue debris and undigested vessels) and was washed three times with 25 mL of EHB buffer (EARLE salts, 25 mM HEPES, 1% BSA, 1 mM sodium pyruvate, without glucose, pH 7.45). For morphometric analysis, aliquots of cell suspension were evaluated in an optical microscope (100× magnification) attached to the 1.3 MP digital camera (Moticam 1000—MOTIC). The program Motic-Images Plus 2.0 was used to measure the area cell cross-section, from which the average cell radius and thus the cell diameter average in μm; 50 cells were measured in each preparation.

### 2.6. Lipolysis Determination

Lipolysis was assessed under baseline conditions (unstimulated). Isolated adipocytes (1 × 10^6^ cells/mL) were incubated in EARLE/HEPES buffer (20 mM) containing 1% BSA (Bovine Serum Albumin) and glucose (5 mM), pH 7.4. The incubation time was 30 min at 37 °C and, at the end (total volume = 200 mL) was centrifuged in a microcentrifuge refrigerated at 0 °C for 5 min at 7000× *g*. Rates (approximately 120 mL) of the infranate were collected to determine the concentration of glycerol released by cells, by the enzymatic-colorimetric method. A free glycerol determination kit was used (Sigma-Aldrich, St. Louis, MO, USA). Results were expressed in nmol of glycerol released by 10^6^ cells.

### 2.7. Adipokine Measurements

In a six-well culture plate, isolated adipocytes (~8 × 10^5^ cells^−1^) were incubated in Dulbecco’s modified Eagle’s medium containing 10% calf serum and 1% penicillin/streptozotocin at 37 °C under a humid atmosphere of 5% CO_2_ for 30 h. The final concentrations of the adipokines adiponectin, IL-10, IL-6, MCP-1, resistin, and TNF-α from the culture supernatant were determined using specific commercially available DuoSet ELISA kits according to the manufacturer (R&D Systems, Minneapolis, MN, USA; catalog numbers DY1119, DY417, DY1069 and DY 410, respectively). The concentrations of the cytokines are expressed in ng per 10^6^ cells as indicated. A similar procedure was used in our previous studies [17].

### 2.8. Statistical Analysis

The data are expressed as mean ± standard error of the mean (SEM). In the first period, Student’s *t*-test was used, while one-way ANOVA variance analysis, followed by Tukey’s post-test, were used in the second period for comparisons between groups. Prism, version 5.0 (GraphPad Software, Inc., San Diego, CA, USA) was used for analysis. *p <* 0.05 was considered statistically significant.

## 3. Results

### 3.1. Food Ingestion and Body Weight Gain

**Figure 2:**
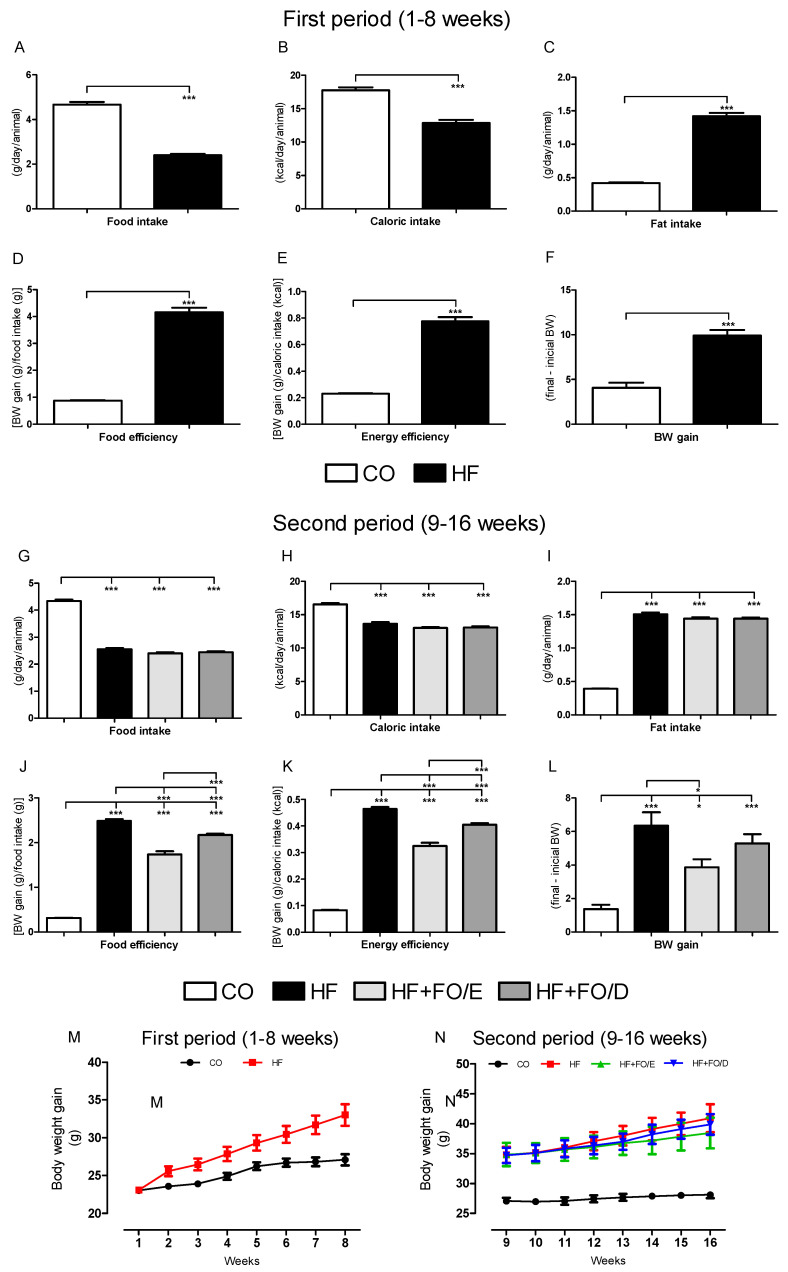
Food intake and body weight (BW) gain of mice fed with control (CO) or high-fat (HF) diet and treated with different compositions of fish oil (FO). During the first period, the animals received only CO or HF diet. During the second period, the diets were maintained and the animals received water (CO and HF groups), or fish oil enriched in either EPA (HF+FO/E group) or DHA (HF+FO/D group) by oral gavage three times per week. Food intake (**A**), caloric intake (**B**), fat intake (**C**), food efficiency (**D**), energy efficiency (**E**) and BW gain (**F**) in the first period. Food intake (**G**), caloric intake (**H**), fat intake (**I**), food efficiency (**J**), energy efficiency (**K**), and BW gain (**L**) in the second period, weekly body mass gain during the first period (**M**), and during the second period (**N**). Mean ± SEM. * *p* < 0.05, *** *p* < 0.001.

During the first eight weeks of the protocol the animals received only the control (CO) or high fat (HF) diets without treatment, and the HF group had a 50% lower food intake than CO group. Furthermore, during this period, the HF group showed a 27.5% reduction in caloric intake (Figure 2A,B), while presenting a higher fat intake, food and energy efficiency, and weight gain (3.5, 4.7, 4.0 and 2.4 fold, respectively) (Figure 2C–F). The evolution of body mass gain during the first 8 weeks can be seen in Figure 2M.

During the second period (9–16 weeks) the animals in the HF group were separated into three different groups: HF, HF+FO/E (5:1 EPA:DHA) and HF+FO/D (5:1 DHA:EPA). There were no statistical differences regarding food, caloric and fat intake between these three groups that received the HF diet supplemented or not with FO (Figure 2G–I).

The HF, HF+FO/E and HF+FO/D groups showed an increase in food efficiency (8.3, 5.7 and 7.3 fold), energy efficiency (5.0, 3.0 and 4.0 fold), and weight gain (4.5, 2.8 and 3.8 fold, respectively) in relation to the CO group. When compared to the HF group, the HF+FO/E and HF+FO/D groups showed a 32% and 12% reduction in food efficiency, and 40% and 20% in energy efficiency, respectively. When comparing only the two FO-treated groups, the HF+FO/D group showed an increase of 33% in food efficiency and 36% in energy efficiency (Figure 2J,K). Only the HF+FO/E group showed a significant reduction in body mass gain (38%) when compared to the HF group (Figure 2L). The weekly body mass gain during second period is shown in Figure 2N.

### 3.2. Glucose and Insulin Tolerance Tests

**Figure 3:**
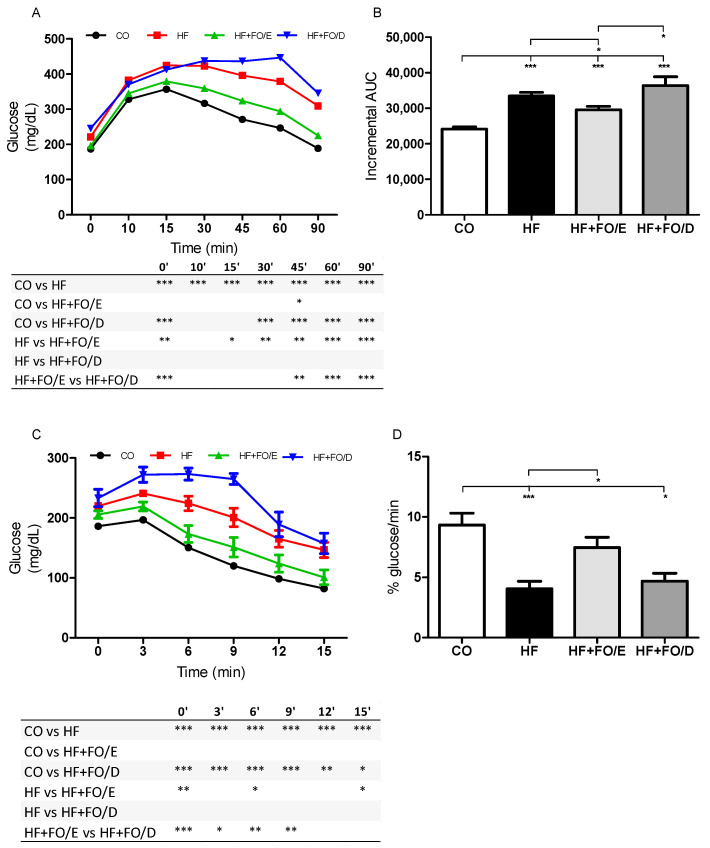
GTT and ITT of mice fed with CO or HF diet and treated with FO. HF+FO/E, HF diet supplemented with FO containing high concentration of EPA. HF+FO/D, HF diet supplemented with FO containing high concentration of DHA. (**A**) GTT, blood glucose concentration versus time after glucose administration. (**B**) Incremental area under the GTT curve. (**C**) ITT, glucose decay curve versus time after insulin administration. (**D**) Glucose disappearance rate for ITT (kITT). FO: fish oil; CO: control diet; HF: high fat diet; HF+FO/E, HF diet supplemented with FO containing high concentration of EPA; HF+FO/D, HF diet supplemented with FO containing high concentration of DHA; GTT: glucose tolerance test; ITT insulin tolerance test. Mean ± SEM. * *p* < 0.05, ** *p* < 0.01, *** *p* < 0.001.

At the end of the protocol the animal’s fasting glycemia (time 0) was evaluated. As expected, the HF diet resulted in hyperglycemia that could only be prevented by the 5:1 EPA:DHA treatment, but not by 5:1 DHA:EPA (Figure 3A). After glucose overload, the HF and HF+FO/D group showed glucose intolerance when compared with the CO group (in 10th and 30th minute, respectively). The HF+FO/E group showed a significant drop in blood glucose after the 15th minute and a progressive reduction of the blood glucose levels similar to that of the CO group (Figure 3A). When the area under the curve was calculated, it was possible to see an increase in glucose intolerance in all groups fed with the HF diet when compared to CO (38.7% HF; 22.7% HF+FO/E; 50.7% HF+FO/D) (Figure 3B). Although, the HF+FO/E group showed an 11,8% reduction of glucose intolerance when compared to the HF and another 23.2% reduction compared to the HD+FO/D group (Figure 3B).

After the insulin overload, we observed lower responsiveness to insulin of the HF and HF+FO/D groups, while that of the HF+FO/E group was similar when compared to CO (Figure 3C). Although all groups fed an HF diet showed a reduction in the glucose disappearance constant (kITT) when compared to CO (56.0% HF; 19.8% HF+FO/E; 49.3% HF+FO/D), an increase of 83% in the HF+FO/E group when compared to the HF was observed (Figure 3D).

### 3.3. Adiposity, Adipocytes Diameter, Liver Weight and Triglycerides, and Total Cholesterol Levels

**Figure 4:**
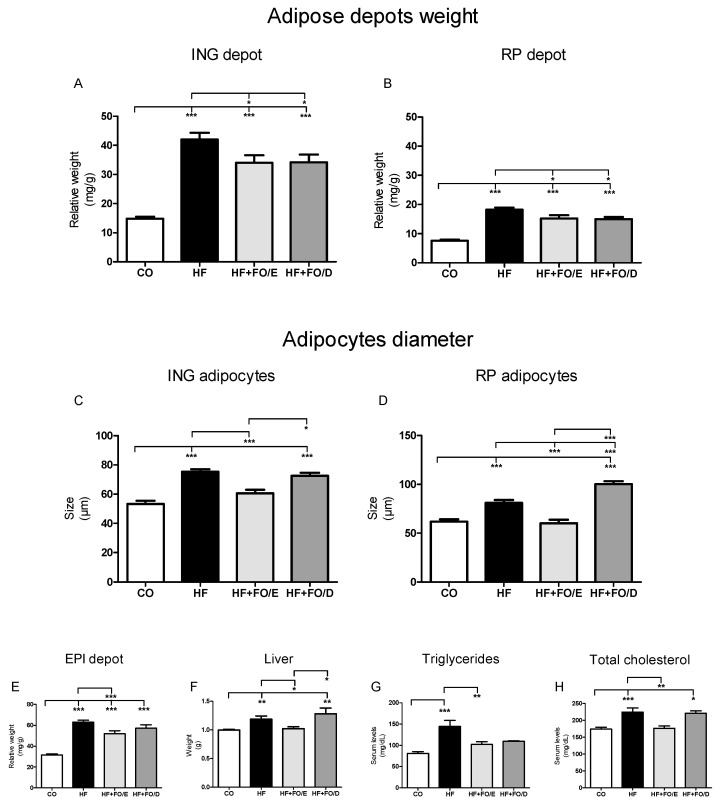
Adiposity and hypertrophy of adipocytes from ING and RP fat depots of mice fed with CO or HF diet and treated with FO. (**A**) relative weight (mg/g BW) of ING fat depot. (**B**) relative weight (mg/g BW) of RP fat depot. (**C**) diameter of ING adipocytes. (**D**) diameter of RP adipocytes. (**E**) relative weight (mg/g BW) of EPI fat depot. (**F**) absolute liver weight. (**G**) serum triglycerides. (**H**) serum total cholesterol. FO: fish oil; CO: control diet; HF: high fat diet; HF+FO/E, HF diet supplemented with FO containing high concentration of EPA; HF+FO/D, HF diet supplemented with FO containing high concentration of DHA; ING: inguinal; RP: retroperitoneal; EPI: epididymal; BW: body weight. Mean ± SEM. * *p* < 0.05, ** *p* < 0.01, *** *p* < 0.001.

After euthanasia, the subcutaneous and visceral adipose depots, and also the liver, were removed and weighed. All groups fed an HF diet (HF, HF+FO/E, and HF+FO/D) showed a significant increase in the mass of the different fat depots when compared to the CO group (ING 2.8, 2.3 and 2.3 fold; RP 2.4, 2.0 and 2.0 fold; EPI 2.0, 1.6 and 1.8 fold respectively). Both omega-3 treatments were able to reduce the mass of ING and RP (19% and 17%, respectively) when groups were compared to HF only (Figure 4A,B), but the 5:1 EPA:DHA supplementation was the only one able to reduce the mass of the EPI depot of the obese animals (Figure 4E). Furthermore, only 5:1 EPA:DHA supplementation was also able to prevent liver weight gain (Figure 4F).

Regarding the diameter of isolated adipocytes, it was observed that the HF and HF+FO/D groups showed significant hypertrophy of cells in the ING (2.5 and 2.2 fold) and RP (2.1 and 3.9 fold, respectively) adipose tissue when compared to the CO group. In the ING depot, the HF+FO/E group adipocytes had an average diameter 42.6% smaller than HF and 34.8% smaller than HF+FO/D adipocytes. The RP adipocytes in the HF+FO/E group were 54.5% smaller when compared to HF. In turn, the HF+FO/D group had significantly larger cells than the HF and HF+FO/E groups (1.8 and 4.0 fold, respectively) (Figure 4C,D).

The treatment with omega-3 was able to prevent the increase in serum triglycerides concentration due to the HF diet (Figure 4G), but only the EPA-enriched supplementation could prevent the total cholesterol increase caused by the HF diet (Figure 4H).

### 3.4. Lipolysis in Isolated Adipocytes

**Figure 5:**
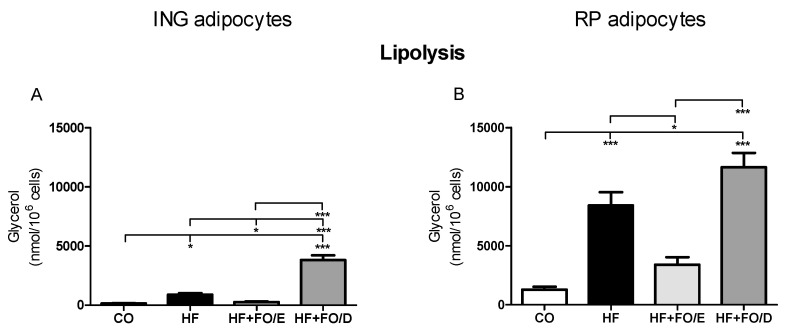
Lipolysis in isolated adipocytes from ING and RP fat depots. (**A**) glycerol released from ING adipocytes, (**B**) glycerol released from RP adipocytes. FO: fish oil; CO: control diet; HF: hifh fat diet; HF+FO/E, HF diet supplemented with FO containing high concentration of EPA; HF+FO/D, HF diet supplemented with FO containing high concentration of DHA; ING: inguinal; RP: retroperitoneal. Mean ± SEM. * *p* < 0.05, *** *p* < 0.001.

The HF and HF+FO/D groups showed greater lipolytic activity in both ING (6.2 and 26.4 fold) and RP adipocytes (6.6 and 9.1 fold, respectively) when compared to their respective CO groups. The HF+FO/E adipocytes showed a reduction in lipolytic activity when compared to the adipocytes from the HF group in ING and RP depots (69.5% and 59.5%, respectively). The HF+FO/D adipocytes were even more lipolytic than the HF+FO/E one in ING and RP depots (14 and 3.4 fold, respectively) (Figure 5A,B).

### 3.5. Secretion of Adipokines by Isolated Adipocytes

**Figure 6:**
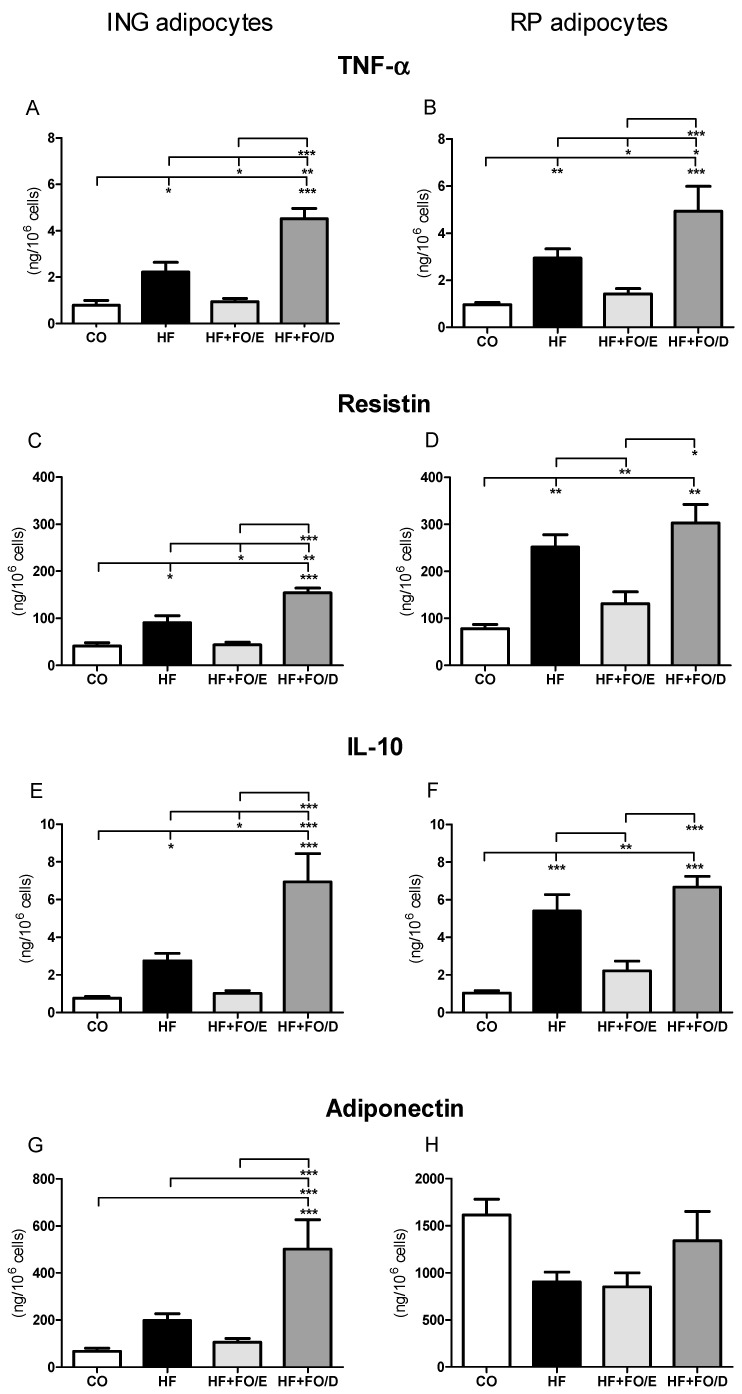
Secretion of cytokines by isolated adipocytes from ING and RP fat depots. (**A**) TNF-α secretion by ING adipocytes. (**B**) TNF-α secretion by RP adipocytes. (**C**) resistin secretion by ING adipocytes. (**D**) resistin secretion by RP adipocytes. (**E**) IL-10 secretion by ING adipocytes. (**F**) IL-10 secretion by ING adipocytes. (**G**) adiponectin secretion by ING adipocytes. (**H**) adiponectin secretion by RP adipocytes. FO: fish oil; CO: control diet; HF: hifh fat diet; HF+FO/E, HF diet supplemented with FO containing high concentration of EPA; HF+FO/D, HF diet supplemented with FO containing high concentration of DHA; ING: inguinal; RP: retroperitoneal; TNF- α: tumor necrosis factor alpha; IL-10: interleukin-10. Mean ± SEM. * *p* < 0.05, ** *p* < 0.01, *** *p* < 0.001.

Regarding the secretion of TNF-α, resistin, and IL-10, it was possible to observe an increase in HF groups both by ING (2.8, 2.2, and 3.4 fold) and by RP adipocytes (2.9, 3.2, and 5.4 fold, respectively) when compared to CO. The HF+FO/E group showed a reversion of these parameters, with a reduction of 59.1% in TNF-α, 52.1% in resistin, and 63% in IL-10 by ING adipocytes, and 51.7%, 47.9%, and 59.3%, respectively, by RP adipocytes (Figure 6A–F).

The HF+FO/D ING adipocytes presented a TNF-α (2.1 fold), resistin (1.7 fold), IL-10 (2.6 fold), and adiponectin (2.5 fold) higher secretion than the HF adipocytes (Figure 6A,C,E,G). In the RP depot, the same was true only for TNF-α (1.7 fold) (Figure 6B). When we compare the HF+FO/D group with the HF+FO/E group, TNF-α, resistin, and IL-10 are higher not only in the ING depot (5.0, 3.6 and 6.9 fold, respectively), but also in the RP one (3.5, 2.3, 3.1, respectively) (Figure 6A–F), and adiponectin (4.7 fold), only by ING adipocytes (Figure 6G).

## 4. Discussion

FO supplementation, containing a combination of EPA and DHA in different proportions, is an important dietary source for omega-3 intake and several studies have shown a potent action of FO in the treatment of obesity and comorbidities [9,16,17,18,19]. However, contradictory results have been reported [28,29,30]. These works mainly differ in terms of type and different omega-3 FA ratios contained in the FO. Herein, we compared the effects on mice of HF diet supplementation with two different proportions of omega-3 FA contained in fish oils available and usually commercialized (5:1 EPA:DHA and the inverse ratio 5:1 DHA:EPA). Our study shows that these different ratios have different impacts on weight gain, glucose and insulin tolerance, circulating cholesterol, and adipose tissue metabolism. Our results bring an important contribution to the elucidation of the different roles of EPA and DHA in vivo in preventing the several metabolic changes caused by an HF diet.

EPA and DHA can elicit biological changes by acting in distinct sites, namely: (1) plasma membrane composition, (2) membrane receptor activation, and (3) nuclear receptor activation. In a model membrane study, it was observed that EPA and DHA occupy distinct locations [31] and that DHA has a greater influence in increasing plasma membrane fluidity of vascular endothelial cells [32]. Moreover, EPA and DHA activate GPR120, an important membrane receptor present in macrophages and adipocytes [12]. GPR120 activation is responsible for pre-adipocytes differentiation [33]. At the gene transcription level, it was shown that EPA and DHA bind to PPARs [34], resulting in increased transcriptional activation [15].

In this study, we show that the two different omega-3 FAs did not affect food, caloric, and fat intake of the supplemented groups (HF+FO/E and HF+FO/D) when compared to the HF group and that they were able to decrease the food and energy efficiency of these groups when compared to HF. EPA-enriched FO showed food and energy efficiency lower than DHA and was able to prevent body weight gain caused by the HF diet. This reduction in body weight may be explained by the fact that, although both FO groups presented the same reduction in the relative weight of ING and RP depots, only the HF+FO/E group showed a decrease in the EPI relative weight and the absolute liver weight when compared to the HF and HF+FO/D group. It is known that the administration of omega-3 FAs associated with an obesogenic diet in rodents can reduce the accumulation of body fat, preventing the development of obesity and associated metabolic disorders [16,17,35,36]. A study that analyzed mice after eight weeks of corn-oil-based HF diet supplemented with 3:1 DHA:EPA showed a reduction in body weight gain of obese mice [37], but not with the same intensity as we have seen with the 5:1 EPA:DHA ratio. Another study involving mice submitted to a three weeks fructose obesogenic diet, followed by five weeks of pure EPA, pure DHA, or 1:1 EPA:DHA supplementation, could not detect a difference in body mass gain between FO-treated groups and control [38]. These two studies [37,38] have also shown that DHA had an important contribution to glycemic control of obese mice, although in our study only the HF+FO/E group showed fasting blood glucose levels, glucose tolerance, and insulin tolerance similar to that of the CO group, and was significantly lower than the HF and HF+FO/D. It is important to note that the quality of the diet is one of the factors most associated with weight gain, hyperglycemia, hypertriglyceridemia, hypertension, decreased HDL, and increased abdominal circumference [39,40,41,42,43,44], and that our protocol is based on a lard-enriched diet.

We show that 5:1 EPA:DHA supplementation, but not 5:1 DHA:EPA, prevented liver weight gain, in agreement with another study of our group which also showed that 5:1 EPA:DHA supplementation in the HF diet maintained all liver parameters similar to that of the control group [45]. Both omega-3 supplementations could prevent the increase in serum triglycerides, but only enriched EPA supplementation prevented total cholesterol increase.

White adipose tissue (WAT) is the main energy reservoir of the mammalian body due to the specialization of adipocytes in storage and mobilization of energy excess obtained from the diet in the form of triacylglycerol (TAG) [46,47]. The WAT mass is determined by the size of adipocytes (hypo or hypertrophy), which varies according to the amount of TAG stored or mobilized, and also by changes in the number of adipocytes (hypo or hyperplasia) [48,49]. As recently reported in our previous work, the 5:1 EPA:DHA ratio was able to decrease the mass of subcutaneous, ING, and visceral, RP, regions, as well as completely reverse the cellular hypertrophy of both depots from obese animals induced by HF diet [16]. In the present work, we observed that FO containing the inverse ratio, 5:1 DHA:EPA, acted similarly in reducing adipose depots mass but not in preventing the adipocyte hypertrophy, in which values were similar to the HF group in ING, and even larger than the HF group in RP adipocytes, meaning that the treatment with FO rich in DHA triggered greater hypertrophy of visceral adipocytes. The reduced adipocyte volume of the HF+FO/E group may be the result of an increase in tissue hyperplasia. It was described as an increase in proliferation of Adipose-derived Stem Cell (AdSC) isolated from ING WAT of obese mice supplemented with 5:1 EPA:DHA [45]. Besides, treating control AdSCs with pure EPA led to an increase in both cell proliferation and differentiation, while pure DHA could only increase cell differentiation [45].

The distribution of adipose tissue depots is an important factor for the risks arising from obesity. This disorder results in dysfunction of WAT and is characterized by hypertrophy of adipocytes, alteration of the profile of immune cells present in the stroma, and in secretion of adipokines [50,51]. Furthermore, WAT from obese individuals has less capacity to expand the capillary network surrounding adipocytes, resulting in hypoxia and cellular necrosis, which contributes to increased macrophage infiltration and low-grade inflammation [52]. In this study, we show the capacity of the EPA-enriched supplementation to prevent the changes in the adipocytes secretion of inflammatory cytokines, which was not detected in the HF+FO/D group. This may reflect the fact that EPA can increase VEGF-A adipocytes secretion in vitro by activating not only GPR120, but also by inducing PPARγ binding to a PPAR-response element in the regulatory region of the *VEGF-A* gene [53]. This could lead to better tissue perfusion than DHA, which has shown a lower PPARγ affinity than EPA [15].

Corroborating the literature, we observed larger adipocytes in the visceral adipose depot with higher lipolytic activity compared to the subcutaneous depot, in all groups of animals. The HF diet led to a significant increase in both tissue mass and adipocyte size and, consequently, to an increase in lipolysis. Higher lipolysis results in a greater release of free FA and increased deposition and accumulation of these lipids in other organs, such as the heart, liver, and pancreas causing lipotoxic effects [21,54]. Herein, FO rich in DHA exacerbated the lipolytic response in both adipose depots, triggering an increase in lipolysis even greater than that presented by the HF group. On the other hand, FO rich in EPA completely reversed the lipolysis in both ING and RP adipocytes. We have addressed the impact of FO (5:1 EPA:DHA ratio) in the liver lipid profile and morphology. The treatment of obese animals with FO rich in EPA was effective in decreasing nonalcoholic steatohepatitis (NASH) and stimulated adipogenesis [45]. The EPA effects in decreasing lipolysis and inducing adipogenesis in WAT corroborate the beneficial effects on reversing liver steatosis.

Herein, FO rich in EPA completely reversed the lipolysis in both ING and RP adipocytes. On the other hand, FO rich in DHA exacerbated the lipolytic response in both adipose depots, triggering an increase in lipolysis even greater than that presented by the HF group.

In addition to impaired metabolic functions, hypertrophied cells have an altered secretory pattern, resulting in increased secretion of pro-inflammatory cytokines [55,56,57,58]. It is known that chronic low-grade inflammation courses with obesity and states of insulin resistance and emerges as an important factor that activates lipolysis in adipocytes [59,60]. TNF-α secreted in WAT induces intracellular lipolysis [61], while IL-6 appears to increase the lipolytic response to the adrenergic stimulus [62].

A study using adipocytes in culture showed that EPA neutralizes the effects of TNF-α and IL-6 on HSL-mediated lipolysis [63]. In our study, we saw a significant increase in the expression of TNF-α, resistin, and IL-10 by adipocytes isolated from ING and RP depots, as a consequence of the HF diet-induced obesity. This increase was completely reversed by treatment with FO rich in EPA. On the other hand, the FO rich in DHA did not decrease the levels of these cytokines, and still induced higher values than the HF group in the secretion of TNF-α by both ING and RP adipocytes, and also in the secretion of resistin and IL-10 by ING adipocytes. An evaluation of obese humans presenting low-grade systemic inflammation after nine weeks of supplementation with 2.7 g/day of EPA or DHA showed that, compared to the control group, EPA reduced IL-6, LDL cholesterol, and TAG [64]. However, except for IL-6, DHA further reduced TAG and LDL cholesterol, as well as CRP, IL-18, TNF-α, and total cholesterol, and increased adiponectin, which corroborates the interesting increase in adiponectin observed in this study only in the HF+FO/D group. It is important to emphasize that adiponectin is the major adipose-derived insulin-sensitizing hormone.

In another study evaluating the effect of EPA associated with HF diet, it was shown that EPA improves inflammatory parameters by reducing MCP-1 and the recruitment of macrophages [65]. This same group evaluated the gonadal WAT cellularity, where the group that received an HF diet for six weeks and EPA for another five weeks, added to the diet, showed no reduction in body mass, thus showing the effect of EPA regardless of obesity [65]. The analysis of the epididymal WAT in an obesity state suggested that the expression of pro and anti-inflammatory genes are increased together during the expansion of WAT in mice receiving an HF diet [66], justifying that the balance of both cytokines is regulated by the energy supply in WAT, and thus the increase in anti-inflammatory aims to balance the activities of pro-inflammatory cytokines. 

In agreement with the present and other works that have shown independent effects of DHA and EPA [38,67,68,69], Mozaffarian and Wu (2012) [70] suggest that EPA and DHA have both shared and complimentary benefits, although purified EPA, but not DHA, reduced the risk of nonfatal coronary syndromes in one large clinical trial.

Finally, when 8-week-old male C57Bl/6J mice were submitted to a 16-week HF diet, despite their lower food intake, they developed obesity, insulin resistance, and an increase in weight of several adipose depots, with hypertrophic and dysfunctional adipocytes in ING and RP WAT. The FO rich in EPA proved to be effective in reversing these parameters, while the FO rich in DHA reversed only the gain of ING and RP adipose depot mass of animals. Thus, the ratio of the different omega-3 FA present in FO capsules available commercially may or may not have effective results for improving obesity and its complications, as well as in other specific diseases.

## Figures and Tables

**Table 1:** Amount of fat present in fish oils.

Amount of Fat per Gram	Fish Oil EPA:DHA	Fish Oil DHA:EPA
Total fat	1.00	1.00
Saturated fat	0.00	0.00
Trans fat	0.00	0.00
Monounsaturated fat	0.00	0.13
Polyunsaturated fat	0.90	0.67
EPA	0.55	0.10
DHA	0.10	0.50
Cholesterol	0.00	0.00

EPA: eicosapentaenoic acid; DHA: docosahexaenoic acid.

## Data Availability

Not applicable.
